# Movement adds bite to the evolutionary morphology of mammalian teeth

**DOI:** 10.1186/1741-7007-10-69

**Published:** 2012-08-16

**Authors:** P David Polly

**Affiliations:** 1 Departments of Geological Sciences, Biology, and Anthropology, Indiana University, 1001 E. 10th Street, Bloomington, Indiana 47401, USA

**Keywords:** dental occlusion, evolutionary constraints, morphological integration, complexity, orientation patch counts

## Abstract

Selection and constraints put limits on morphological evolution. Mammalian teeth are no exception, and the need for them to meet precisely exerts exacting constraints on a staggering array of developmental and functional factors that must be integrated to maintain their performance as they evolve. A study in *BMC Evolutionary Biology *demonstrates that mandibular movement is an important component of this integration, and one that should not be neglected in the quantitiative study of the evolution of tooth morphology.

See research article http://www.biomedcentral.com/1471-2148/12/146/

## Commentary

**Figure 1:**
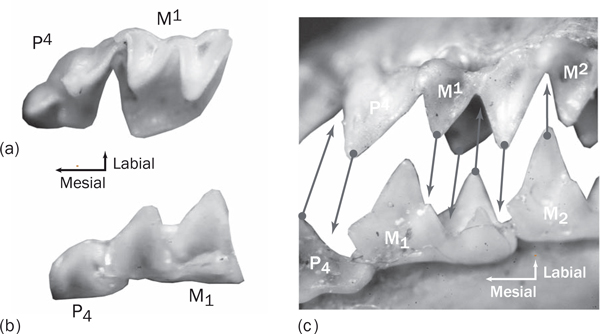
**Asymmetry in form and complex occlusal interlocking between upper and lower mammalian cheek teeth**. **(a,b) **The upper (a) and lower (b) fourth premolar and first molar of the bat *Pipistrellus *in occlusal view showing the difference in morphology between the maxillary and mandibular dentitions. Despite their differences, these teeth interlock precisely along a complex, complementary series of surfaces, as shown by **(c) **the teeth of the bat *Barbastellus *in their functional orientation. The arrows show corresponding points that slide into contact as the animal chews. In mammals with this type of occlusion, there is only one possible angle through which the lower teeth can move into occlusion with the uppers. Coordination among the forms of the individual teeth, the sizes of the upper and lower jaw, the position and structure of the jaw joint, and the vectors of movements of the several muscles of mastication are required for these mammals to be able to eat. These factors have different developmental genetic controls, and the constraining force of integrative stabilizing selection must be strong, yet this functional complex evolves quickly enough that even a non-specialist can see morphological differences in the teeth of these two con-familial, moth-eating specialists.

Despite the magnificent morphological diversity of life, limits are imposed on phenotypic evolution by many factors: curbs from lack of genetic and developmental variability, boundaries from physical laws such as gravity, restrictions imposed by functionally integrated morphology, exclusions by competitive interactions with other species, barriers imposed by climate and resources, and constraints arising from the cumulative specializations inherited from ancestors. No system so forthrightly illustrates rapid evolvability in the face of such limitations as dental occlusion in mammals [1]. Teeth evolve so quickly that mammal species can usually be distinguished from their molars alone [2], yet the tight interlocking between upper and lower teeth required for proper masticatory function creates a seemingly contradictory need for both strong developmental genetic control and extreme genetic malleability. Even though the morphologies of upper and lower teeth are different, their occluding surfaces must be precisely complementary for effective chewing, especially in dentitions that are specialized for slicing (Figure 1). In many mammals, there is one and only one path that the lower teeth can follow into proper occlusion during chewing [3]. In this issue of *BMC Evolutionary Biology*, Smits and Evans demonstrate quantitatively that the direction of that path is a significant factor integrating the functional complexity of upper and lower teeth, implying that many of the limiting factors relevant to the evolution of the mammalian dentition may be correlated with the vector describing that path [4].

To appreciate the significance of Smits' and Evans' findings, one must understand the scale and complexity of integration necessary to maintain a functional occlusion. Anyone who has experienced orthodontic treatment knows how precise occlusion must be to eat comfortably and effectively, and how easily malocclusions can develop. The selective importance of occlusion in mammals has been demonstrated by loss of fitness in older female Sifakas, a species of lemur, whose teeth are too worn to obtain proper nutrition to sustain lactation during lean seasons [5]. Occlusion in many mammals creates such a fidelity of shape between upper and lower teeth that palaeontologists can determine whether isolated tooth fossils belong to the same species by how well they fit together [6]. Indeed, the mismatch between upper and lower teeth of different species is so phylogenetically scaled that it has almost clock-like properties [7]. The closeness of the occlusal match is substantial enough that one might hypothesize a simple, unifying developmental genetic control that maintains occlusion in the face of population and evolutionary variation. That hypothesis would be wrong.

One obstacle to the development of occlusion is that the relative lengths of the upper and lower jaws must be kept in sync. The developmental signaling centers for the maxillary and mandibular arches are independent, with only a few factors to help coordinate their developmental growth [8]. Incompatible growth of mandible and maxilla is a major cause of human malocclusions that develop in teens and young adults, and mismatch of upper and lower jaws is characteristic of many dog breeds, such as pugs and bulldogs.

A second obstacle to the development of occlusion is that the morphology of the upper and lower teeth must independently be formed before the teeth ever come into contact. Mammalian teeth do not develop in the mouth, but in the bone of the mandible and maxilla below the gingiva. Their shape is formed first in soft tissue by by interaction between epithelium and neural crest mesoderm that creates a model of the shape of the future crown, which is then mineralized from the cusp tips to the base [1]. Only after the mineralized tooth crown is formed does it erupt into the mouth and come into occlusion with its counterpart. In most species tooth development starts *in utero *and continues until young adulthood when the last molars erupt into the mouth. While there is some opportunity in all mammals for the spacing between teeth to be adjusted post-eruption and the opportunity in certain herbivorous and omnivorous species for wear to reshape the teeth for a better fit, the upper and lower teeth must otherwise be pre-formed with exactly the right shape to fit together before they ever join the tooth row. If the upper and lower teeth were mirror images of one another, the mechanism for coordinating the upper and lower teeth would seem easier to imagine, but the two rows of teeth have differentiated morphologies (Figure 1), despite sharing a common cascade of developmental genetic interactions that establishes their basic shape and having some features that are affected by the same [1,9]. Interestingly, mammalian clades that have evolutionarily abandoned complex occlusion, such as toothed whales, seals, and pangolins, have lost the differentiation in morphology between upper and lower teeth. The production of differentiated teeth that fit tightly together is clearly complicated, which may be why mammals and a few dinosaur taxa are the only major groups to have evolved complexly occluding dentitions, most other vertebrates having either simple, conical homodont dentitions or no teeth at all.

The many competing factors that must be coordinated to maintain proper occlusion during evolutionary transformations is evident, but establishing the relative importance of the factors is more difficult because the dental system and its occlusal and evolutionary dynamics are difficult to quantify. One of the challenges for Smits and Evans in their study was quantifying the morphology of the upper and lower teeth [4]. The three-dimensional complexity of the teeth make traditional morphometrics, which are based on a series of size measurements, inadequate for the task, and the differences in structure of the upper and lower teeth make geometric morphometrics, which are applied to biologically or geometrically homologous points, difficult to apply. Smits and Evans used a recently developed quantification of the functional component of tooth form, the orientation patch count (OPC), to overcome the challenge. The OPC describes the number of discrete faces on the surface of the tooth crown, estimated from three-dimensional scans using algorithms similar to those commonly used to measure the slopes of mountains, hills, and valleys in geographic information systems (GIS) [10]. Teeth with lots of small surfaces with different orientations have a high OPC, whereas teeth with a few large surfaces in the same orientation have a low OPC. This simple measure describes the same features that functional morphologists long ago identified as important components of dietary adaptation, namely the size, number, and orientation of dental wear facets [3]. Herbivores, which must shred and pulp the hard-to-digest cellulose cell walls of vegetation before swallowing, tend to have low crowns with many small facets giving them a high OPC, whereas carnivores, which slice easily digested animal tissues into chunks, tend to have high crowns with only a few vertically oriented facets giving them a low OPC. The OPC index is objective in that it is measured algorithmically, it is numerical, which allows the functionally important aspects of complex dental morphology to be analyzed quantitatively, and it can be applied to teeth of any morphology, which allows upper and lower teeth or teeth from different clades to be compared. Smits and Evans used OPC as a measure of the functional complexity, where more patches means higher complexity, for comparing upper and lower teeth. Because the morphology of the two tooth rows is different, their OPCs, and hence complexity, are also different despite their common role in mastication.

Smits and Evans found that the direction of mandibular movement is an important contributor to the correlation between upper and lower teeth [4]. The OPC of the lower teeth by themselves was a weak predictor of the OPC of the upper teeth compared to the angle of movement combined with the OPC of the lowers. In other words, the functional morphology of upper teeth is not only related to the morphology of the lowers, but also to the direction that the lowers move across the uppers. That direction is controlled by the position and shape of the jaw joint, and the points of attachment of the three major muscles of mastication, adding even more complexity to the factors controlling the integration of occlusion. Most importantly, Smits and Evans' results indicate that mandibular kinetics is a central factor of the dental system and needs to be included to better understand the morphological integration of occlusion in individual species and the correlated changes in morphology, anatomy, and developmental dynamics that are associated with evolution of dental morphology. Smits' and Evans' work opens many transformative questions. Are the most rapid evolutionary changes in tooth morphology ones that do not require a change in the direction of mandibular movement? Or do all evolutionary changes in dental morphology involve changes in mandibular direction? Are there developmental genetic factors unique to mammals that link the formation of joints, muscles, and teeth to coordinate the control of the path of mastication? Should major transitions in dental evolution be reconceptualized in terms of changes in mandibular kinetics? The complicated evolution of the integrated mammalian dentition offers a rich system to address many questions of broad relevance to evolutionary biology.
